# Supplementary material for: A Process and Rubric for a Group to Review the Quality of a Medical Education Course/Clerkship
Source: MedEdPORTAL. 2020 Jun 18;16:10911. doi: 10.15766/mep_2374-8265.10911 (PMC7331966; doi:10.15766/mep_2374-8265.10911)
Supplement: Supplementary file 1 — Checklist of Materials.docxCourse Director Questions.docxCourse Review Report Rubric.docxReview Meeting Guide.docxCourse Review Process Survey.docx [file _Educational_Summary_Report_10911.pdf › _Educational_Summary_Report_10911.pdf]

MedEdPORTAL® The AAMC Journal of Teaching and Learning Resources

Original Publication Open Access

A Process and Rubric for a Group to Review the Quality of a Medical Education Course/Clerkship

Kathryn B. Moore, PhD*, Rachel Bonnett, Jorie M. Colbert-Getz, PhD, MS

*Corresponding Author: kathryn.moore@neuro.utah.edu

Abstract

Introduction: Reviewing elements of a curriculum, such as courses and clerkships in medical school, is an essential part of the quality improvement process. Yet there is a gap in the literature in terms of actual rubrics for evaluating course quality in medical schools. **Methods:** This resource describes a course review process and rubric to evaluate course quality: A subcommittee of faculty members and students evaluates goals, content and delivery, assessment, feedback to students, grading, and student feedback for each course with the rubric. Course directors, Curriculum Committee members, and Curriculum Evaluation Subcommittee members were surveyed on their perception of the process. **Results:** A large majority of Curriculum Committee and Curriculum Evaluation Subcommittee members agreed that the review process was objective (100%), provided an evaluation of course quality (>95%), helped identify areas of improvement/strengths (>91%) and issues/concerns in the curriculum (>95%), helped them become more familiar with the curriculum (>90%), and was a catalyst for changes in a course (>77%). Course/clerkship directors had less agreement that the course review process was a catalyst for curriculum changes (46%) and that the process helped identify areas of improvement for a course (62%). **Discussion:** This curriculum evaluation process provides a resource for other institutions to use and/or modify for their own course evaluation process. All stakeholders in the review process agreed that the evaluation process was successful in identifying areas that worked and did not work in courses.

Keywords
Course Evaluation, Review Process, Curricular Evaluation, Quality Improvement, Program Evaluation

Educational Objectives

By using this process, MD program stakeholders will be able to:

1. Identify key areas for evaluating the quality of a course.
2. Critically appraise whether a course meets, exceeds, or is below expectations in key areas.
3. Evaluate the applicability of the process for reviewing the quality of a course as relevant to local program needs.

Introduction

In higher education, learning is most often considered a dynamic process; as a consequence, the evaluation process may be best viewed as a cycle of action research designed to meet the changing needs of students, educators, and institutions.[1]

Citation:
Moore KB, Bonnett R, Colbert-Getz JM. A process and rubric for a group to review the quality of a medical education course/clerkship. *MedEdPORTAL*. 2020;16:10911.
https://doi.org/10.15766/mep_2374-8265.10911

Additionally, an effective evaluation process should be able to drive evidence-based change in courses and the curriculum as a whole.[2] The challenge for medical schools is how best to evaluate curricular effectiveness while providing educators with informative feedback necessary to improve teaching and learning effectiveness. Thus, an effective course evaluation process should be able to integrate the needs of on-the-ground educators (course directors and teaching faculty) and the larger institutional goals. In other words, a review process must be designed to satisfy all stakeholders in the educational process. For example, reviewing the quality of a course has to be important to those conducting the review so that they devote sufficient time, has to provide administrators with information about course quality to help them decide when to act, and, most importantly, has to motivate course directors and teaching faculty to implement changes as needed.

There are many types of program evaluation methods, and most have in common an analysis of process, structure, and outcomes to determine quality. There are currently no guidelines for ideal types of process, structure, and outcomes that would

Copyright © 2020 Moore et al. This is an open-access publication distributed under the terms of the Creative Commons Attribution license.

indicate a medical school course is high quality. Although there are studies that provide rationales and guidance for enhancing course evaluation and/or overall curriculum evaluation in higher education,[3-5] as well as one in medical education,[6] there remains a gap in the literature in terms of actual rubrics for evaluating course quality in medical schools. Furthermore, medical education curricula are different than higher education programs and courses, so the elements deemed important to review in college courses may not work for medical schools. To date, there have been no publications in *MedEdPORTAL* on a course or clerkship review process. The goals of our resource are to (1) describe a review process for medical school courses and clerkships and (2) evaluate the effectiveness of that process.

Methods

This course review process was implemented for an undergraduate medical education program in the United States and meant to apply to courses, clerkships, and subinternships. Henceforth, course will refer to course, clerkship, or subinternship. Targeted users are other US medical schools accredited by the Liaison Committee on Medical Education (LCME). There is no prerequisite knowledge needed by users of this resource, but familiarity with LCME standards related to courses and evaluations may be helpful.

Curriculum Evaluation Subcommittee Structure and Membership

The Curriculum Evaluation Subcommittee reported to the School of Medicine Curriculum Committee. The subcommittee included approximately 10 faculty members and was chaired by the assistant dean of education quality improvement, who was also a faculty member. To ensure a fair and balanced evaluation process, we desired a spectrum of representatives from clinical and foundational science faculty who had a range of experience in the curriculum, from seeing only a few students on electives each year to directing a course. This intentional mix of member experiences ensured that those delivering the curriculum were not the same people evaluating the curriculum. However, we found having a few faculty members with a high level of involvement in the curriculum (e.g., course director) was beneficial to discussions. Faculty members applied to join the subcommittee with a brief statement of interest and were elected by the faculty body to serve a 3-year term that could be renewed once. All members committed to attending at least half of all meetings in a year. Additionally, to capture the student point of view of a course after completion, two to three students were elected each academic year by the student body to serve on the subcommittee. Their medical school year level varied by the courses being reviewed. Students were not able to be members if they were in the prior or current year of the curriculum being reviewed (e.g., if all year 2 courses were being reviewed, only third- and fourth-year medical students were eligible to serve on the subcommittee). The education quality improvement manager (hereafter referred to as program manager) supported the Curriculum Evaluation Subcommittee by collecting all course information in a centralized place for subcommittee members to review.

The Curriculum Evaluation Subcommittee formally reviewed each course/clerkship and required experience of the curriculum at our school every 3 years. This resulted in 12-14 review meetings each year. It took a staff member approximately 2 hours to gather information for each course/clerkship review, approximately 1.5 hours for each subcommittee member to review materials, 1.5 hours for each review meeting, and an additional 30 minutes to write up and finalize the review report. The course director time commitment varied between 1 and 1.5 hours.

Course Review Process

The course review time line is shown in the Figure, and the list of information reviewed is provided in Appendix A.

The program manager collected materials 4 weeks in advance of the meeting. The program manager had access to shared drives and online course modules where all items were stored. The program manager created several documents for the subcommittee from course documentation to aid the subcommittee's prep time and to allow the members to compare categories year to year. Course directors and course coordinators were contacted about materials only if items could not be found in shared drives and online course modules. Course directors were sent a copy of the collected materials 2 weeks in advance so that they had a record of what the subcommittee would be reviewing. Course directors also received a list of questions 2 weeks in advance of the meeting. They typically spent 30 minutes to 1 hour preparing to answer the questions at the meeting and reviewing materials that had been gathered by the program manager. If course directors could not attend the meeting, they submitted their answers to the questions in writing. Total estimated time for course directors in this review process was 1-1.5 hours.

Subcommittee members then reviewed the course information 2 weeks prior to the scheduled review meeting. During the review meeting, the course director answered questions from the subcommittee for 30 minutes. Most of these questions were standard and are provided in Appendix B, but subcommittee members could (and often did) ask additional questions based

Copyright © 2020 Moore et al. This is an open-access publication distributed under the terms of the Creative Commons Attribution license.

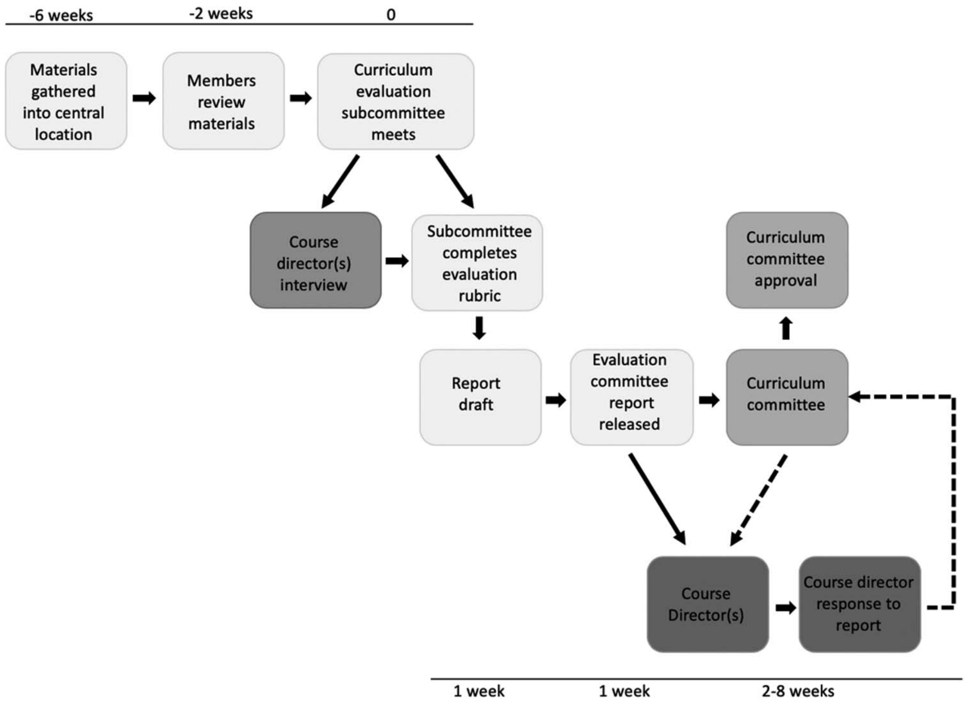

Figure. Course review time line.

on the material reviewed. Responses by the course director were recorded by the chair and, if necessary, were verified by reviewing the other evidence residing in the box folder. After the questions had been answered, the course/clerkship director left, and subcommittee members independently rated each element of the review with a rubric (Appendix C) based on their prior reading of course information and the course director's responses to the questions. The specific areas evaluated were course goals, content and delivery, assessment, feedback to students, grading, and student feedback. The subcommittee discussed any element rating for which there was not 100% agreement until consensus was reached. Refer to Appendix D for a meeting guide outlining what the chair and members do during the meeting.

Once the rubric was completed, the subcommittee identified aspects of the course that were working well plus any changes required based on elements rated as being below expectations. The subcommittee also could note recommendations for other aspects of the course that did not fall below expectations but should be monitored or might need improvement. Any required change had to be addressed by the course director, and he or she could determine if specific recommendations warranted being addressed. The assistant dean of education quality improvement presented the review report to the Curriculum Committee for discussion and approval. Then, the course director received the approved review report and was asked to provide a response to any required changes specified in it within 30 days. The response was presented to the Curriculum Committee for discussion and approval.

Course Review Rubric Development

We used program theory to develop our review rubric. First, we outlined the program to be evaluated, which was the quality of individual courses in a curriculum. Then, we identified elements (rubric items) for measuring quality. Due to the limited literature on measures of course quality in medical education, rubric items were developed locally. A PhD educator identified aspects that targeted course quality/compliance from the 2016-2017 LCME Data Collection Instrument (DCI). The PhD educator had experience in survey design, and she created the rubric scale and wording of each review element. Finally, to help prioritize the quality elements and determine what evidence was necessary for to meet or exceed expectations, the associate and assistant deans of the curriculum reviewed the rubric before it was reviewed a final time by faculty members serving on the Curriculum Committee. Thus, there was evidence of content validity based on the expertise of those creating and revising the rubric and the use of the LCME DCI for mapping of items. Additionally, there was a mix of elements relating to structure,

Copyright © 2020 Moore et al. This is an open-access publication distributed under the terms of the Creative Commons Attribution license.

process, and outcomes of a course, key features of most program evaluations methods for determining quality.

We then determined the materials to gather that would help subcommittee members rate each element. Some elements required course director input, so we decided to create a standard set of course director questions. We limited the questions to those that would be enhanced by course director input (e.g., “Describe the process for selecting what content is taught and how often you review the content”) and avoided questions that could be objectively answered with course information (e.g., “Do all course sessions have learning objectives?”).

Evaluation of Course Review Process

We have been using the course review process described here since spring 2016. At the 3-year mark (spring 2019), we decided to evaluate the process with a survey. In April 2019, the course review process survey (Appendix E) was sent via email to three groups: course directors who had had their course evaluated in the past 2 years, current Curriculum Committee members, and current Curriculum Evaluation Subcommittee members. The survey was developed by a faculty member who had served in all three group roles and a PhD educator with experience in survey writing. Survey items were based on goals of the evaluation process and a literature review of course evaluation in higher education. Respondents provided their agreement on a strongly agree, agree, disagree, strongly disagree scale about the process being objective, providing an evaluation of course quality, helping identify areas of improvement and strengths for a course, identifying issues/concerns in the curriculum, and being a catalyst for changes at a course and curriculum level. Respondents also indicated whether the process had helped them become more familiar with the curriculum. The percentages of agree and strongly agree ratings were computed for each group. Course directors also indicated whether the subcommittee had a good understanding of their courses on a strongly agree, agree, disagree, strongly disagree scale, and the frequencies of ratings were computed. Curriculum Evaluation Subcommittee members also rated the usefulness of each resource (e.g., syllabus) for reviewing/evaluating the quality of a course as very helpful, somewhat helpful, or not at all helpful. Frequencies for each rating by item were computed. Finally, everyone was asked, “What are you not getting out of the course review process that you would like?” Comments were analyzed for themes.

Results

The response rates for the course review process survey were 67% (20 out of 30) for Curriculum Committee members, 73% (11 out of 15) for Curriculum Evaluation Subcommittee members, and 80% (16 out of 20) for course directors. The Table provides the number and percentage of agree/strongly agree ratings for each aspect of the review process by the three stakeholder groups. A large majority of stakeholders (>70%) agreed or strongly agreed with all but three items. Course directors had less than 70% agree or strongly agree ratings for (1) “The course review process helps identify areas of improvement for a course,” (2) “The course review process is a catalyst for curriculum-level changes,” and (3) “The course review process helps [them] become more familiar with the curriculum.” The majority of course directors strongly agreed (13%, $n = 2$) or agreed (56%, $n = 9$) with the statement “The Curriculum Evaluation Subcommittee had a good understanding of my course.”

All Curriculum Evaluation Subcommittee members (100%, $N = 11$) found seven of nine resources to be somewhat to very helpful for evaluating the quality of a course: course goals, end-of-course student feedback, student feedback of individual instructors, assessment performance, grades, discussion with the course director, and review rubric. The two exceptions were the syllabus (81%, $n = 9$) and session objectives (91%, $n = 9$).

Positive themes from the comments suggested that the review process worked well for identifying compliance items such as

Table. Number and Percentage of Agree/Strongly Agree Ratings by Three Stakeholder Groups (“Not Able to Rate” Responses Omitted)

Statement	Curriculum Committee Members: No. (%)	Curriculum Evaluation Subcommittee Members: No. (%)	Course Directors: No. (%)
The course review process is objective.	20 (100%)	11 (100%)	15 (94%)
The course review process provides an evaluation of course quality.	19 (95%)	11 (100%)	12 (74%)
The course review process helps identify areas of improvement for a course.	20 (100%)	10 (91%)	10 (62%)
The course review process helps identify strengths of a course.	20 (100%)	11 (100%)	12 (75%)
The course review process helps identify issues/concerns across the curriculum.	19 (95%)	11 (100%)	12 (75%)
The course review process is a catalyst for changes in a course.	14 (70%)	11 (100%)	12 (75%)
The course review process is a catalyst for curriculum-level changes.	15 (77%)	8 (77%)	6 (46%)
The course review process helps me become more familiar with the curriculum.	18 (90%)	11 (100%)	4 (25%)

Copyright © 2020 Moore et al. This is an open-access publication distributed under the terms of the Creative Commons Attribution license.

having objectives aligned with assessments and seeing the big picture of the curriculum. Given how the survey item was worded, there were two main themes for areas of improvement from comments. First, the review process should focus more on what worked well within a course rather than on what did not work well.

- “More focus on positive/what is working metrics.”
- “The set-up of the review process is mostly designed for negative comments (which are appreciated), but not very informative as to what is going well.”

Second, the review process should rely less on student input and find other ways to evaluate quality, such as peer feedback.

- “A better balance between student satisfaction and a critical look level of challenge/expectations would be ideal.”
- “I think peer feedback and observation are something that is lacking in the course review process and would be very helpful.”

Discussion

The curriculum evaluation process described here provides a rubric for other institutions to use and/or modify for their own course evaluation process. The Curriculum Evaluation Subcommittee members endorsed the rubric for course evaluation, indicating that it provided clear expectations and utilized data to support committee recommendations. Use of the rubric also removed opinion from formal decision making. Providing details in the survey’s comment section was essential for any item rated below expectations and helped us maintain consistency across courses. The comments also helped us gain credibility with the Curriculum Committee as it has not argued against any of our required changes or recommendations in the past 3 years.

Course directors, Curriculum Evaluation Subcommittee members, and Curriculum Committee members all agreed that the evaluation process was helpful in identifying areas that worked and areas that did not work. Course directors, however, saw the process as working better for incremental course improvements rather than driving larger curricular changes. For example, the review could identify whether there were course goals mapped to competencies, note trends in faculty performance, and summarize student satisfaction. That said, the process has driven some curricular-level change over the past 3 years. For example, an issue was identified within a single subinternship course that led to global discussions about monitoring of faculty appointments for adjunct faculty.

In the past 3 years, we have made the following changes to our review process. Prior to academic year 2017-2018, we asked for volunteers only from School of Medicine basic science and clinical departments, and there were no term limits. In academic year 2017-2018, we started to have the faculty and student body elect members for the subcommittee, and we also set up terms, primarily for LCME governance purposes. Also starting in academic year 2017-2018, we provided a detailed description of each type of assessment used in a course to the committee members as not all members were familiar with our entire system of assessment. In academic year 2018-2019, we started the consensus process where each member independently rated elements before discussion. This was done because the chair was concerned that not all members felt comfortable speaking up/voicing their thoughts. In general, the subcommittee did not struggle to come to a consensus for items in the rubric, although there was often debate concerning what constituted an innovation within a course. Better curricular-wide definitions for course innovation would likely reduce/alleviate this issue. We also changed the order of the report at the request of the Curriculum Committee. The Curriculum Committee thought course directors would be more welcoming of the entire report if it started with aspects of the course that were working well. We used to have an item about the course director having a clear vision for future iterations of the course, but we had difficulty measuring this, so we took it off the report since it was not specifically related to an LCME standard. Finally, after the first year of this review process, we started to notice areas of improvement that were not necessarily the responsibility of the course directors but instead pertained to the curriculum administration. These areas usually related to policies and practices across an entire year or more of the curriculum. Thus, we added an item requesting recommendations for curriculum leadership to the rubric.

We have learned that a critical aspect of the success of the evaluation process is the creation of a centralized location for review documents. This streamlining allows the members to focus on the actual review process rather than spending their valuable time tracking down documents from various learning management pages, electronic storage folders, and so on. The timing of review is another important aspect of this process. We have found that reviewing each course every 3 years is optimal as it allows course directors time to make and implement changes and to assess whether those changes have been successful. This 3-year review cycle also allows the subcommittee to better separate minor, transitory problems within a course from more problematic downward trends. When team-based learning was first implemented into the curriculum,

Copyright © 2020 Moore et al. This is an open-access publication distributed under the terms of the Creative Commons Attribution license.

many courses saw a dip in student satisfaction. Evaluation on the 3-year cycle showed that this dip was most likely due to the time it took both faculty and students to buy in to a novel teaching method. However, it is important to note that if there are major changes in course leadership or elsewhere, an additional review can be done out of cycle. For example, one of our courses replaced both course directors simultaneously, and subsequently, the course has been reviewed more frequently to allow the directors and Curriculum Committee to better assess the outcomes of this substantial leadership change. A change in the review cycle can be requested by the Curriculum Evaluation Subcommittee, the Curriculum Committee, and/or the course directors. The Curriculum Committee ultimately approves the cycle and schedule of reviews annually. Additionally, a summary report of each course is provided to the Curriculum Committee to monitor course quality between formal reviews. The exact timing of each review is a component other schools will need to consider based upon their specific curricular and course director needs.

The composition of the subcommittee is something schools might also want to modify to meet their needs. At our school, we seek members willing to devote time to the process instead of balancing representatives from basic science departments and clinical departments, although it is critical to have both faculty types on the subcommittee. Faculty subcommittee members agree that it is also important to have students on the subcommittee, particularly third- and fourth-years, to provide both a longitudinal perspective that can be missing from immediate student feedback on a course and a closer view/perspective of the curriculum. Our school does compensate the subcommittee members with a small stipend sent to their department (∃3,000/academic year), but this has not been cited as a reason for continued engagement. Overall, most members see the role as a valuable component of the curriculum and consider themselves part of the curriculum team.

A few limitations of the process should be noted. The review process does not directly evaluate the quality of content, although this is done somewhat indirectly by reviewing student feedback. In the future, we plan on adding more detail about content quality and will enlist expert faculty to help with that part of the review process. Other schools might want to consider how best to incorporate content quality reviews, since adding more faculty input can be time intensive and potentially resource intensive. The review process does not specifically address learning environment, since our institution has been addressing this issue on an institutional level. However, we have found that learning environment often comes up in course evaluations (e.g., in data about mistreatment rates and student comments). Although we do not rate courses formally on their learning environment, our process has illuminated several issues about the learning environment which have then been communicated to the Curriculum Committee in the course reports. Finally, although the review process is useful for driving smaller, tactical curricular changes, the current format may not motivate course directors to improve their courses. It might be helpful, as a reviewer pointed out, to have course directors reflect on such questions as the following: What is the best thing about your course? What are you most worried about? What are you most interested in addressing the next time you offer this course/clerkship?

Based upon our 3 years of experience with this rubric and our survey results, we have concluded that the addition of a peer feedback component, a question on learning environment, a more specific place for course directors to reflect on faculty preparation and the successes and challenges of their course, and more emphasis on positive outcomes of courses may be helpful for motivation to trigger more strategic, global changes.[7,8] We are currently testing the addition of a course director reflection component and determining the best way to add peer evaluation that works for the foundational science courses as well as for clerkships.

Appendices

A. Checklist of Materials.docx
B. Course Director Questions.docx
C. Course Review Report Rubric.docx
D. Review Meeting Guide.docx
E. Course Review Process Survey.docx

All appendices are peer reviewed as integral parts of the Original Publication.

Kathryn B. Moore, PhD: Professor, Department of Neurobiology and Anatomy, University of Utah School of Medicine

Rachel Bonnett: Manager, Education Quality Improvement, University of Utah School of Medicine

Jorie M. Colbert-Getz, PhD, MS: Assistant Dean of Education Quality Improvement, University of Utah School of Medicine; Associate Professor, Department of Internal Medicine, University of Utah School of Medicine

Copyright © 2020 Moore et al. This is an open-access publication distributed under the terms of the Creative Commons Attribution license.

Disclosures
None to report.

Funding/Support
None to report.

Ethical Approval
Reported as not applicable.

References

1. Harris L, Driscoll P, Lewis M, Matthews L, Russell C, Cumming S. Implementing curriculum evaluation: case study of a generic undergraduate degree in health sciences. *Assess Eval High Educ*. 2010;35(4):477-490. https://doi.org/10.1080/02602930902862883
2. Harvey L, Newton L. Transforming quality evaluation: moving on. In: Westerheijden DF, Stensaker B, Rosa MJ, eds. *Quality Assurance in Higher Education*. Springer Nature; 2007:225-245.
3. Skelton J, West KP, Zeff T. Phase 1 of a comprehensive course review process: program innovation. *J Dent Educ*. 2002;66(3):405-413. https://doi.org/10.1002/j.0022-0337.2002.66.3.tb03519.x
4. Stevenson TL, Hornsby LB, Phillippe HM, Kelley K, McDonough S. A quality improvement course review of advanced pharmacy practice experiences. *Am J Pharm Educ*. 2011;75(6):116. https://doi.org/10.5688/ajpe756116
5. Persky AM, Joyner PU, Cox WC. Development of a course review process. *Am J Pharm Educ*. 2012;76(7):130. https://doi.org/10.5688/ajpe767130
6. Goldman EF, Swayze SS, Swinehart SE, Schroth WS. Effecting curricular change through comprehensive course assessment: using structure and process to change outcomes. *Acad Med*. 2012;87(3):300-307. https://doi.org/10.1097/ACM.0b013e318244739c
7. Chism NV. *Peer Review of Teaching: A Sourcebook*. 2nd ed. Anker Publishing: 2007.
8. Horowitz S, Van Eyck S, Albanese M. Successful peer review of courses: a case study. *Acad Med*. 1998;73(3):266-271. https://doi.org/10.1097/00001888-199803000-00014

Received: July 24, 2019
Accepted: November 29, 2019
Published: June 18, 2020

Copyright © 2020 Moore et al. This is an open-access publication distributed under the terms of the Creative Commons Attribution license.
