# Supplementary material for: A Process and Rubric for a Group to Review the Quality of a Medical Education Course/Clerkship
Source: MedEdPORTAL. 2020 Jun 18;16:10911. doi: 10.15766/mep_2374-8265.10911 (PMC7331966; doi:10.15766/mep_2374-8265.10911)
Supplement: Supplementary file 1 — Checklist of Materials.docxCourse Director Questions.docxCourse Review Report Rubric.docxReview Meeting Guide.docxCourse Review Process Survey.docx [file mep_2374-8265.10911-s001.zip › A. Checklist of Materials.docx]

**Appendix A**

**Checklist of Materials to Gather for Course Review**

- Syllabi from prior 3 Academic Years
- Session objectives from most recent completed Academic Year
- Percentage of instructional modalities used in course from prior 3 Academic Years
- List and Description of assessments from most recent completed Academic Year
- Alignment of course goals with program objectives / Entrustable professional activity (EPA) and assessment
- Assessment performance
  - Historical – mean and standard deviation for past 3 Academic Years on similar assessments
  - By program objectives / EPA for current academic year
- Numerical and Letter Grade Distribution for past 3 Academic Years
- Number of days for all students to receive final grade for past 3 Academic Years
- End-of-Course student feedback rating and comment reports and student feedback for all instructors in course
