# Supplementary material for: A Process and Rubric for a Group to Review the Quality of a Medical Education Course/Clerkship
Source: MedEdPORTAL. 2020 Jun 18;16:10911. doi: 10.15766/mep_2374-8265.10911 (PMC7331966; doi:10.15766/mep_2374-8265.10911)
Supplement: Supplementary file 1 — Checklist of Materials.docxCourse Director Questions.docxCourse Review Report Rubric.docxReview Meeting Guide.docxCourse Review Process Survey.docx [file mep_2374-8265.10911-s001.zip › B. Course Director Questions.docx]

**Appendix B**

**Course Director Questions**

1. Describe the process for selecting what content is taught and how often you review the content.

2. What steps are taken to coordinate content within the course and with other parts of the curriculum to minimize omissions and unplanned redundancies?

3. Clerkships Only: How do you ensure that students are meeting the patient criteria and if not, what do you do?

4. Do you think the major assessments and grading are truly measuring students’ abilities?

5. Describe your mechanism for reviewing and sharing student feedback, including the data reviewed, and individuals or groups responsible for reviewing the information.

6. How do you address students concerns?

7. What happens when faculty receive consistently poor evaluations?

8. What changes, if any, have you made to the course based on prior assessment performance and/or student feedback?

9. Do you feel you have adequate resources (including coordinator support) to direct your course?

*Note that subcommittee members may have additional questions specific to your course*
