# Supplementary material for: A Process and Rubric for a Group to Review the Quality of a Medical Education Course/Clerkship
Source: MedEdPORTAL. 2020 Jun 18;16:10911. doi: 10.15766/mep_2374-8265.10911 (PMC7331966; doi:10.15766/mep_2374-8265.10911)
Supplement: Supplementary file 1 — Checklist of Materials.docxCourse Director Questions.docxCourse Review Report Rubric.docxReview Meeting Guide.docxCourse Review Process Survey.docx [file mep_2374-8265.10911-s001.zip › C. Course Review Report Rubric.docx]

**Appendix C**

**Course Review Report Rubric**

| Course: |  | Date of Review: |  |
| --- | --- | --- | --- |
| Director(s): |  | Curriculum Committee Approval Date: |  |

| **Element** | **Exceeds Expectations (EE)** | **Meets**  **Expectations (ME)** | **Below**  **Expectations** | **Comments** |
| --- | --- | --- | --- | --- |
| **Course Goals, Outcomes, and Objectives** | | | | |
| **The course goals support the SOM program objectives and/or Entrustable Professional Activities** | n/a as this is a yes or no item | Each goal is accurately aligned with a SOM program objective or EPA | Does not meet expectations criteria |  |
| **The course goals are assessed** | n/a as this is a yes or no item | Each goal is appropriately aligned with one or more assessments | Does not meet expectations criteria |  |
| **There are written objectives for each education session** | n/a as this is a yes or no item | There are objectives for all sessions | Does not meet expectations criteria |  |
| **Course Content and Delivery** | | | | |
| **Educational methods are appropriate for the stated goals and objectives** | Balance of instructional methods is appropriate to achieve goals and objectives, and novel/creative and effective methods are utilized | Balance of instructional methods is appropriate to achieve goals and objectives | Does not meet expectations criteria |  |
| **Resources at each site support an effective learning environment (Clinical course only)** | There is sufficient space for didactics, studying, and call with minimal computer / internet issues and an appropriate number of learners on service at all sites | There is sufficient space for didactics, studying, and call with minimal computer / internet issues and an appropriate number of learners on service at most sites, with improvement plans in place for sites that do not meet the criteria | Does not meet expectations criteria |  |
| **Course includes content that integrates well within the curriculum** | Course director(s) routinely coordinates with other courses to minimize omissions and unplanned redundancies | Course director(s) has coordinated with other courses to minimize omissions and unplanned redundancies, but this was done quite some time ago | Does not meet expectations criteria |  |
| **Course Assessment, Feedback, and Grading** | | | | |
| **Each assessment measures the aligned course goal and program objective** | ME and assessment scores indicate if students have achieved the course goal and program objective | It is clear that each assessment is intended to measure the aligned course goal and program objective | Does not meet expectations criteria |  |
| **Formative assessment and feedback are provided to students** | n/a as this is a yes or no item | Students have the chance to demonstrate their performance and receive feedback with the use of formative assessment | Does not meet expectations criteria |  |
| **Grading is transparent and fair** | n/a as this is a yes or no item | Grading process is clearly defined in the syllabus and all evidence indicates that this process was followed including students receiving assessment scores/narrative feedback and final grade by the specified time. | Does not meet expectations criteria |  |
| **Student Feedback** | | | | |
| **Students are satisfied with their experience** | At least 90% of students rate the course quality AND organization as excellent or good. | At least 80% of students rate the course quality AND organization as excellent or good. | Does not meet expectations criteria |  |
| **Student concerns have been addressed** | Examples of student concerns, changes made, and outcomes provided | Examples provided, but unclear yet if change will be effective | Does not meet expectations criteria |  |

**Aspects of this course/clerkship that are working well:**

**Required changes for next year:**

**Recommendation(s) for improvement:**

**Recommendations for Curriculum Leadership:**
