# Supplementary material for: A Process and Rubric for a Group to Review the Quality of a Medical Education Course/Clerkship
Source: MedEdPORTAL. 2020 Jun 18;16:10911. doi: 10.15766/mep_2374-8265.10911 (PMC7331966; doi:10.15766/mep_2374-8265.10911)
Supplement: Supplementary file 1 — Checklist of Materials.docxCourse Director Questions.docxCourse Review Report Rubric.docxReview Meeting Guide.docxCourse Review Process Survey.docx [file mep_2374-8265.10911-s001.zip › D. Review Meeting Guide.docx]

**Review Meeting Guide**

**Curriculum Evaluation Subcommittee Chair**

- Ensures the standard course director questions and any and follow-up/additional questions are answered fully by the course director
- Ensures the course director and members stay on task during the question-answering section of the meeting to avoid running over time
- Ensures there are no unresolved issues or questions by members prior to completing the review rubric
- Facilitates the group process for completing the review rubric by ensuring:
  - Relevant data or evidence for an element is displayed/discussed

Everyone independently rates each element

- Everyone indicates his or her rating by show of hands
- There is consensus building discussion for any element rating not with 100% agreement by members
- The final rating is recorded
- This process is repeated until all rubric elements are rated
- Members identify areas of concern based on any elements rated below expectations
- Areas with required changes or recommendations are recorded
- Members identify strengths of the course
- Ares of strength are recorded
- Members identify for any relevant phase or curricular level concerns to note in the report
- The group stays on task to avoid running over time

**Curriculum Evaluation Subcommittee Member**

- Participates in the questions-answering section by asking follow-up/additional questions as needed to the course director
- Participates in the group process for completing the review rubric by:
  - Independently rating each element when asked on a copy of the rubric
  - Discussing rationale / evidence for any element rating not in 100% agreement
  - Respectfully listening to other members providing their rationale / evidence
  - Discussing areas of concern and areas of strength
