# Supplementary material for: A Process and Rubric for a Group to Review the Quality of a Medical Education Course/Clerkship
Source: MedEdPORTAL. 2020 Jun 18;16:10911. doi: 10.15766/mep_2374-8265.10911 (PMC7331966; doi:10.15766/mep_2374-8265.10911)
Supplement: Supplementary file 1 — Checklist of Materials.docxCourse Director Questions.docxCourse Review Report Rubric.docxReview Meeting Guide.docxCourse Review Process Survey.docx [file mep_2374-8265.10911-s001.zip › E. Course Review Process Survey.docx]

Course Review Process Survey

To be completed electronically

Instructions/Opening Statement Reviewing courses is a LCME accreditation need, but how we review courses is up to us as a school.  We would like the process to be informative, result in efficient change, and not be a burden to course directors.  Thus, we are gathering feedback to determine where to make improvements to the course review process so that is does more than meet accreditation.

1. Indicate your agreement with the following aspects of the review process:

|  | Strongly  Agree | Agree | Disagree | Strongly Disagree | Not able to rate |
| --- | --- | --- | --- | --- | --- |
| The course review process is objective |  |  |  |  |  |
| The course review process provides an evaluation of course quality |  |  |  |  |  |
| The course review process helps identify areas of improvements for a course |  |  |  |  |  |
| The course review process helps identify strengths of a course |  |  |  |  |  |
| The course review process helps identify issues / concerns across the curriculum |  |  |  |  |  |
| The course review process is a catalyst for changes in a course |  |  |  |  |  |
| The course review process is a catalyst for curriculum-level changes |  |  |  |  |  |
| The course review process helps me become more familiar with the MD program curriculum |  |  |  |  |  |

2. What are you not getting out of the course review process that you would like?
3. **Course Directors ONLY**

|  | Strongly  Agree | Agree | Disagree | Strongly Disagree | Not able to rate |
| --- | --- | --- | --- | --- | --- |
| The Curriculum Evaluation Sub-committee has a good understanding of my course: |  |  |  |  |  |

4. **Curriculum Evaluation Subcommittee Members ONLY**

Please rate how helpful each aspect is in reviewing / evaluating the quality of a course:

|  | Very Helpful | Somewhat Helpful | Not at all Helpful |
| --- | --- | --- | --- |
| Syllabus |  |  |  |
| Course Goals |  |  |  |
| Session Objectives |  |  |  |
| End-of-Course Student Feedback |  |  |  |
| Student Feedback of Individual Instructors |  |  |  |
| Student Assessment Performance |  |  |  |
| Final Course Grades |  |  |  |
| Discussion with Course Director |  |  |  |
| The review rubric |  |  |  |
